# Mast Cells and Gastrointestinal Dysmotility in the Cystic Fibrosis Mouse

**DOI:** 10.1371/journal.pone.0004283

**Published:** 2009-01-27

**Authors:** Robert C. De Lisle, Lauren Meldi, Eileen Roach, Maureen Flynn, Racquel Sewell

**Affiliations:** Anatomy & Cell Biology, University of Kansas School of Medicine, Kansas City, Kansas, United States of America; Charité-Universitätsmedizin Berlin, Germany

## Abstract

**Background:**

Cystic fibrosis (CF) has many effects on the gastrointestinal tract and a common problem in this disease is poor nutrition. In the CF mouse there is an innate immune response with a large influx of mast cells into the muscularis externa of the small intestine and gastrointestinal dysmotility. The aim of this study was to evaluate the potential role of mast cells in gastrointestinal dysmotility using the CF mouse (*Cftr^tm1UNC^*, *Cftr* knockout).

**Methodology:**

Wild type (WT) and CF mice were treated for 3 weeks with mast cell stabilizing drugs (ketotifen, cromolyn, doxantrazole) or were treated acutely with a mast cell activator (compound 48/80). Gastrointestinal transit was measured using gavage of a fluorescent tracer.

**Results:**

In CF mice gastric emptying at 20 min post-gavage did not differ from WT, but was significantly less than in WT at 90 min post-gavage. Gastric emptying was significantly increased in WT mice by doxantrazole, but none of the mast cell stabilizers had any significant effect on gastric emptying in CF mice. Mast cell activation significantly enhanced gastric emptying in WT mice but not in CF mice. Small intestinal transit was significantly less in CF mice as compared to WT. Of the mast cell stabilizers, only doxantrazole significantly affected small intestinal transit in WT mice and none had any effect in CF mice. Mast cell activation resulted in a small but significant increase in small intestinal transit in CF mice but not WT mice.

**Conclusions:**

The results indicate that mast cells are not involved in gastrointestinal dysmotility but their activation can stimulate small intestinal transit in cystic fibrosis.

## Introduction

Cystic fibrosis (CF) is an autosomal recessive genetic disease caused by mutations in the *CFTR* (cystic fibrosis transmembrane conductance regulator) gene. CFTR is a cAMP-regulated chloride channel that in the intestine is mainly expressed in the apical plasma membrane of crypt cells. In the absence of CFTR, fluid secretion into the intestinal lumen is of low volume and is deficient in bicarbonate, which impairs neutralization of gastric acid. The poorly-hydrated acidic luminal environment is believed to cause accumulation of mucus, which fosters bacterial overgrowth of the CF small intestine [1].

Hallmarks of CF in affected epithelial organs, including the intestine, are accumulation of excessive mucus, microbial infection, and inflammation. Inflammation is known to affect gastrointestinal motility patterns [2], [3]. Inflammation of the human CF intestine has been reported [4]–[6] but, because such studies use indirect measures or require invasive methods, there is not much detailed information. Inflammation of the CF intestine may be a response to bacterial overgrowth, which is reported to occur in up to half of CF patients [7], [8]. Consistent with intestinal inflammation, gastrointestinal dysmotility has been reported to be frequent in CF patients [8]–[14].

The *Cftr* knockout mouse (*Cftr^tm1UNC^*, CF mouse) small intestine exhibits many of the same changes that occur in CF patients, including excessive mucus accumulation, impaired intestinal transit, bacterial overgrowth, and an inflammatory response [15], [16]. Inflammation in the CF mouse intestine has characteristics of an innate response that includes an influx of mast cells, mainly into the muscularis externa tissue [17].

Mast cells have been implicated in various conditions where gastrointestinal motility is altered [18]. Mast cells can stimulate motility during acute intestinal infection with pathogenic bacteria [19]. Mast cells produce histamine and serotonin, both of which can stimulate enteric smooth muscle contraction [20]. In other conditions mast cells are associated with impaired motility, often due to deleterious effects of mast cell proteases on the interstitial cells of Cajal [21] or on the enteric nervous system [2], [18], [22]–[24]. For example, using *in vitro* co-cultures of enteric nerves with peritoneal mast cells, it was shown that mast cells damage the nerves [25]. Inclusion of the mast cell stabilizer doxantrazole in the culture system prevented nerve cell damage demonstrating the importance of mast cell activity.

Because the CF mouse small intestine has impaired transit and a dramatic increase in mast cells in the muscularis, we hypothesized that the mast cells may play a role in slowed gastrointestinal transit in these animals. To test this, we used various agents to stabilize or stimulate mast cells, and measured the effects on gastrointestinal transit *in vivo*.

## Methods

### Ethics Statement

All animal procedures were approved by the Institutional Animal Care and Use Committee.

### Materials

Unless otherwise specified, all chemicals were obtained from Sigma-Aldrich (St. Louis, MO, USA).

### Animals

*Cftr*(+/−) mice *(Cftr^tm1UNC^*) were originally obtained from the Jackson Laboratory (Bar Harbor, ME, USA). As previously described, the mice were bred to be congenic on the C57BL/6J background [26]. *Cftr*(+/−) mice were bred to obtain wild type (WT) [*Cftr*(+/+)] and CF [*Cftr*(−/−)] mice. *Cftr*(+/−) mice, which are phenotypically indistinguishable from WT, were used occasionally as WT. Mice of both sexes were used between 6–7 weeks of age and no gender-related differences in the measured parameters were observed. To prevent lethal intestinal obstruction, all mice were fed *ad libitum* a complete elemental liquid diet (Peptamen; Nestle, Deerfield, IL, USA) [27]. The following mast cell stabilizers were administered at doses derived from published work, by addition to the liquid diet for three weeks: ketotifen (1 mg/kg-day) [28], cromolyn (10 mg/kg-day) [29], [30]; doxantrazole (10 mg/kg-day) [31]. The mast cell stimulator compound 48/80 was administered acutely by i.p. injection of 0.75 mg/kg body weight [32] 30 min before gavage of the motility tracer (see below).

### Measurement of gastric emptying and gastrointestinal transit

Gastric emptying and transit were measured as previously described [15]. Briefly, mice were fasted overnight with free access to water. In the morning between 8–9 AM, mice were gavaged with 100 µl 25 mg/ml rhodamine dextran (MW = 70000). After 20 min, the mice were killed and the gastrointestinal tract was divided into stomach, 10 equal segments of small intestine, and large intestine. The stomach and intestinal segments were flushed and the concentration of the tracer was determined fluorometrically in a Synergy HT microplate reader (BioTek, Winooski, Vermont, USA). Gastric emptying was calculated as the percent of tracer remaining in the stomach relative to the total amount recovered from the stomach and intestine. For measurement of small intestinal transit the fluorescence data were expressed per segment relative to the total in the intestine. For some experiments the post-gavage time was 90 min.

### Mast cell histochemistry

Tissue was fixed with 4% paraformaldehyde and paraffin sections were prepared. Sections were stained with toluidine blue which stains all mature mast cells [33], [34] by binding to serglycin proteoglycans in their secretory granules [35]. Slides were imaged on a Nikon Diaphot microscope equipped with a SPOT RT-KE digital camera (Diagnostic Instruments, Sterling Heights, MI, USA). Mast cells were counted in images of the stained slides by an observer unaware of the sample identities. Tissue areas were calculated using NIH Image J software.

### Statistical analysis

Statistical significance was determined by ANOVA with a post-hoc Fisher's least significant difference test (Systat, Chicago, IL, USA). A *P*-value of <0.05 was considered significant.

## Results

**Figure 1:**
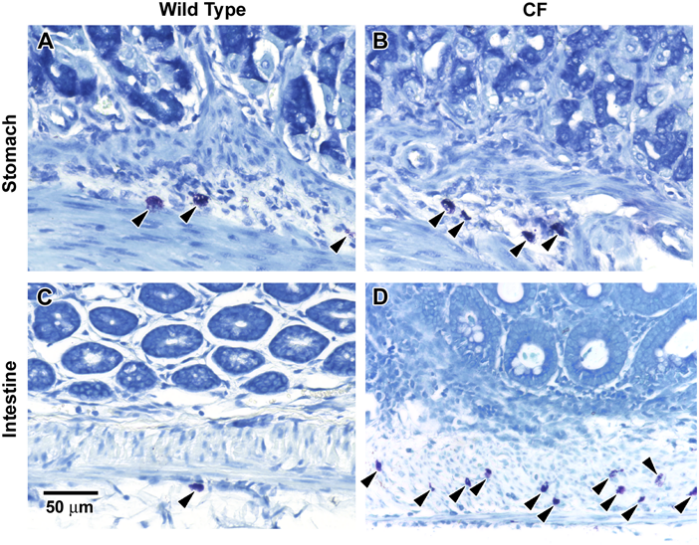
Histochemistry for mast cells in WT and CF mouse stomach and small intestine. Sections were stained with toluidine blue and mast cells identified by their metachromatic staining. (A) Wild type stomach; (B) CF Stomach; (C) Wild type small intestine; (D) CF small intestine. Arrowheads indicate mast cells. Representative from 7 WT and 7 CF stomachs.

**Figure 2:**
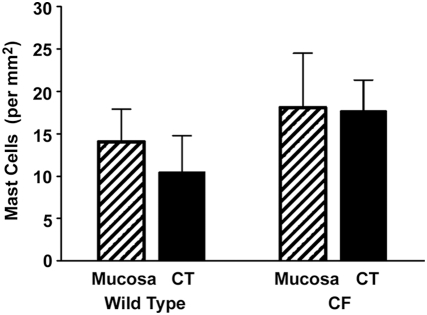
Quantitation of mast cells in stomach of WT and CF mice. There were no significant differences in the numbers of mast cells per unit area in the CF stomach as compared to WT, in either mucosa or connective tissue (CT, which included the muscularis externa). Data are means ± SEM. (n = 7 WT, 7 CF stomachs)

The aim of this study was to test the role of mast cells in gastrointestinal dysmotility in the CF mouse. We have previously shown that the CF mouse has many more mast cells in the small intestine as compared to wild type (WT) mice [17]. It was not known if the numbers of mast cells in the CF mouse stomach also differed, so we first examined this in wild type (WT) and CF stomachs. Mast cells were readily observed in both WT and CF stomach tissues (Fig. 1A and B, respectively). There was no significant difference in the numbers of mucosal or connective tissue/muscularis externa mast cells comparing WT and CF stomachs (Fig. 2). The density of mast cells we measured in the stomach agrees well with previous reports on healthy mice [33].

Our previous work showed that in the small intestine of the CF mouse in comparison to WT, there are 50–100 fold more mast cells, principally in the muscularis externa [17] (Fig. 1C). In contrast, mast cells are rare in the WT intestine, consistent with other reports [33], [36], [37]. The occasional mast cells that were observed in the WT intestine were mostly in the peritoneal space (Fig. 1D) and none were in the muscualris externa.

**Figure 3:**
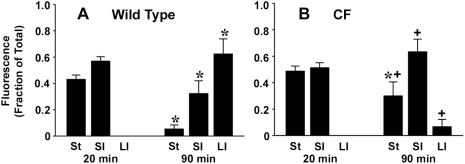
Distribution of rhodamine dextran along the gastrointestinal tract of WT and CF mice at 20 min and 90 min post-gavage. Overnight fasted mice were gavaged with a bolus of rhodamine dextran (see Methods). At either 20 min or 90 min post-gavage the mice were sacrificed and the distribution of the fluorescence in the stomach (St), small intestine (SI) and large intestine (LI) was measured and expressed relative to the total. (A) WT mice (n = 28 mice 20 min post-gavage, n = 8 mice 90 min post-gavage); (B) CF mice (n = 14 mice 20 min post-gavage, n = 7 mice 90 min post-gavage). Data are means ± SEM. (*) *P*<0.05 comparing 20 min vs 90 min data for the same organ and genotype of mice. (+) *P*<0.05 comparing CF vs WT for the same organ and post-gavage time.

**Figure 4:**
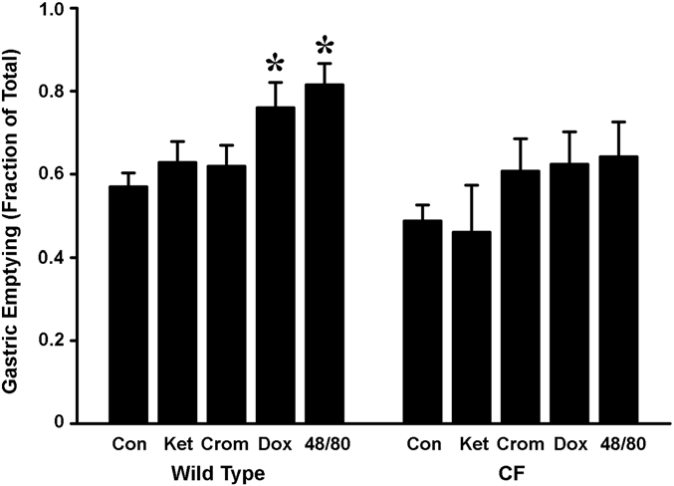
Gastric emptying in WT and CF mice and effect of mast cell agents. Overnight fasted mice were gavaged with a bolus of rhodamine dextran (see Methods). At 20 min post-gavage the mice were sacrificed and the distribution of the fluorescence in the stomach and small intestine was measured. Gastric emptying is expressed as fraction of total fluorescence which has exited the stomach in the 20 min post-gavage period. Con = control (n = 28 WT, n = 14 CF); Ket = ketotifen, 3 week treatment (n = 10 WT, n = 6 CF); Crom = cromolyn 3 week treatment (n = 11 WT, n = 7 CF); Dox = doxantrazole 3 week treatment (n = 5 WT , n = 8 CF); 48/80 = compound 48/80 acute i.p. injection (n = 4 WT, n = 6 CF). Data are means ± SEM. (*) *P*<0.05 vs Control of the same genotype. Data for the control WT and CF mice include values from previous work [1].

To measure gastric emptying and intestinal transit, mice were fasted overnight and in the morning they were gavaged with rhodamine dextran, a non-absorbable tracer. Twenty or 90 min later, the distribution of the fluorescent tracer was measured in the stomach, small intestine, and large intestine. At 20 min post-gavage in untreated control mice gastric emptying was somewhat less in CF mice as compared to WT, but the difference was not statistically significant (Fig. 3, 4). In both WT and CF mice, at 20 min post-gavage the remainder of the tracer was in the small intestine with none in the large intestine (Fig. 3). When the post-gavage period was extended to 90 min, WT mice showed significant decreases of the tracer in the stomach and small intestine, and an increase in the large intestine (Fig. 3A), as compared to the 20 min WT data. In the CF mice at 90 min post-gavage, there was significantly less tracer in the stomach as compared to the 20 min CF data (Fig. 3B). In comparison to WT, the CF mice had significantly more tracer in the stomach and small intestine, and less in the large intestine (Fig. 3B) at 90 min post-gavage. We chose to use 20 min post-gavage for the remainder of the experiments. At this time point, the distribution of tracer in the gastrointestinal tract would allow detection of changes in gastric emptying or improved small intestinal transit comparing WT to CF mice after treatment with the mast cell active agents.

To assess the effects of mast cells on gastric emptying and small intestinal transit we treated mice with various mast cell stabilizers. There are several drugs known to stabilize mast cells, and there is some specificity as to whether mucosal or connective tissue mast cells are affected. Because of the uncertainty of the phenotype of mast cells in the CF intestine (see Discussion) and which drugs would act on which type of mast cells, we used three different drugs.

**Figure 5:**
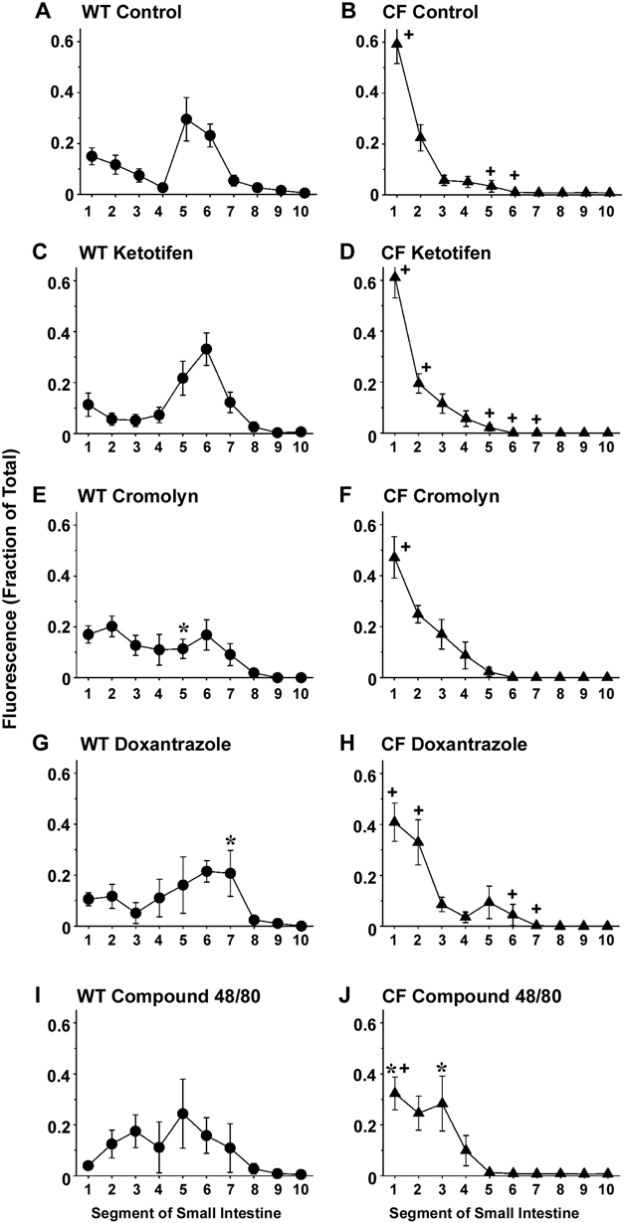
Transit of rhodamine dextran in the small intestine and effect of mast cell agents. Overnight fasted mice were gavaged with a bolus of rhodamine dextran (see Methods). At 20 min post-gavage the mice were sacrificed and the distribution of the fluorescence in 10 segments (proximal to distal) of the small intestine was measured and expressed relative to the total fluorescence. (A) WT Control (n = 28); (B) CF Control (n = 14); (C) Ketotifen, 3 week treatment of WT (n = 10); (D) Ketotifen, 3 week treatment of CF (n = 6); (E) Cromolyn, 3 week treatment of WT (n = 11); (F) Cromolyn, 3 week treatment of CF (n = 7); (G) Doxantrazole, 3 week treatment of WT (n = 5); (H) Doxantrazole, 3 week treatment of CF (n = 8); (I) Compound 48/80, acute i.p. injection of WT (n = 4); (J) Compound 48/80, acute i.p. injection of CF (n = 6). Data are means ± SEM. (*) *P*<0.05 vs control, same genotype and same intestinal segment; (+) *P*<0.05 CF vs WT, same segment and treatment group. Data for the control WT and CF mice include values from previous work [1].

The first drug used was ketotifen, which stabilizes connective tissue mast cells and is also a histamine H1 receptor antagonist. Ketotifen treatment for three weeks had no effect on gastric emptying in either WT or CF mice (Fig. 4). Ketotifen also did not affect the distribution of the tracer along the small intestine (small intestinal transit) in either WT or CF mice (Fig. 5C and D).

The second drug used was cromolyn, which stabilizes mucosal mast cells. Cromolyn was without effect on gastric emptying in WT or CF mice (Fig. 4). In WT mice treated with cromolyn, the tracer was more evenly distributed along the length of the small intestine as compared to control WT (Fig. 5E). Cromolyn had no effect on the distribution of tracer in the CF mouse small intestine (Fig. 5F).

Doxantrazole, a drug that targets both connective tissue and mucosal mast cells, was the third one used. There was a significant increase in gastric emptying in WT mice treated with doxantrazole but not in CF mice (Fig. 4). Doxantrazole caused a slight but significant change in the distribution of the tracer in the small intestine of WT mice (Fig. 5G) but the effect was not significant in CF mice (Fig. 5H).

We next tested the effect of stimulation of mast cells by compound 48/80, administered as an i.p. injection 30 min before gavage of the motility tracer. As compared to control, compound 48/80 significantly increased gastric emptying in WT mice (Fig. 4). In CF mice compound 48/80 had a small effect on gastric emptying as compared to control CF mice, but the difference did not reach the level of statistical significance (*P* = 0.08) (Fig. 4). In WT mice treated with compound 48/80, the distribution of the fluorescent tracer in the small intestine was more even than in control WT mice, but the effect was not statistically significant (Fig. 5I). In contrast, mast cell activation caused a significant change in the distribution of the tracer in the CF mouse small intestine (Fig. 5J). Whereas there was little tracer past the second segment of the small intestine in control CF mice, in the compound 48/80 treated CF mice there was significantly less tracer in the first segment and more in the third segment.

## Discussion

In this study we investigated the potential role of mast cells in gastrointestinal dysmotility in the CF mouse model of cystic fibrosis. Whereas healthy WT mice have very few mast cells in the small intestine [33], [37], in contrast to humans where intestinal mast cells are common, there is a dramatic elevation of mast cells in the CF mouse small intestine. This influx of mast cells in the CF small intestine is likely in response to the bacterial overgrowth that occurs. The bacteria that overgrow the CF small intestine are opportunistic and they colonize the mucus that accumulates in the CF intestine [1], [16]. When bacterial overgrowth in CF mice is treated with oral antibiotics, the level of mast cells is reduced to that of control WT mice [16].

Because mast cells have been shown to affect gastrointestinal motility in various conditions, including in the bacterially infected intestine, we hypothesized that mast cells would play an important role in dysmotility in the CF mouse gastrointestinal tract. However, using a variety of mast cell stabilizing drugs, there was no significant improvement in gastric emptying or small intestinal transit in the CF mouse. There was a small but significant increase in small intestinal transit after activation of mast cells with compound 48/80 in CF mice but not in WT mice. This demonstrates that the increased numbers of mast cells in the CF intestine can affect intestinal motility, but their presence alone does not underlie the dysmotility of the CF gut.

Mast cells display tremendous diversity depending on their tissue site and the nature of the inflammation to which they are responding [38], [39]. Two main ways to classify mast cells are based on their tissue localization and on their enzymatic profile. By an earlier Affymetrix GeneChip analysis, all of the 11 mast cell protease genes on the array were called absent in the WT mouse small intestine [17]. This is consistent with the very low numbers of mature mast cells in the healthy mouse small intestine. In the CF mouse intestine several mast cell protease genes were expressed: mast cell protease 1 (*Mcpt1*), mast cell protease 2 (*Mcpt2*), mast cell chymase 2 (*Cma2*, also called *Mcp10*), mast cell carboxypeptidase A3 (*Cpa3*), and mast cell protease-like (*Mcptl*) [17]. There was unmeasurable expression of the other mast cell markers on the arrays of the CF samples [mast cell protease 4 (*Mcpt4*), chymase 1 (*Cma1*, also called *Mcpt5*), trypase β2 (*Tpsb2*, also called *Mcpt6*), and tryptase α/β1 (*Tpsab1*, also called *Mcpt7*)].

Based on tissue localization and protease gene expression, it is not clear how to classify the mast cells in the CF mouse small intestine. These mast cells are predominantly localized to the muscularis externa layer (Fig. 1D), with occasional cells in the submucosa [17]. This localization would classify them as connective tissue mast cells. However, their protease expression profile does not fit the distinct patterns of mucosal vs. connective tissue mast cells (e.g., Mcpt1 and Mcpt2 are regarded as a mucosal mast cell markers in mice, and Cpa3 is connective tissue mast cell marker, but all are expressed in the CF intestine; and whereas Cma1 is a connective tissue marker it is not expressed in the CF intestine) [38]–[41].

Because of the uncertainty of how to classify the mast cells in the CF small intestine, we used a variety of mast cell stabilizers. However, none of the mast cell stabilizers which have activity on the different types of mast cells had a positive effect on gastrointestinal motility in the CF mouse.

In summary, this study shows that mast cell stabilizers were not able to improve gastrointestinal motility in CF mice. There was a small increase in small intestinal transit in CF mice when mast cells were stimulated but this effect was small and transit was still much less than in healthy WT control mice. Thus, unlike other conditions where dysmotility occurs, mast cells are not likely to be a useful therapeutic target to improve gastrointestinal motility in CF.
